# An Insight into the Anxiolytic and Antidepressant-Like Proprieties of *Carum carvi* L. and Their Association with Its Antioxidant Activity

**DOI:** 10.3390/life11030207

**Published:** 2021-03-05

**Authors:** Imane Es-safi, Hamza Mechchate, Amal Amaghnouje, Fatima Zahra Jawhari, Omkulthom Mohamed Al Kamaly, Hamada Imtara, Andriy Grafov, Amina Bari, Dalila Bousta

**Affiliations:** 1 Laboratory of Biotechnology, Environment, Agrifood, and Health, University of Sidi Mohamed Ben Abdellah, FSDM-Fez 30050, Morocco; Imane.essafi1@usmba.ac.ma (I.E.-s.); Amal.amaghnouje@usmba.ac.ma (A.A.); Fatimazahra.jawhari@usmba.ac.ma (F.Z.J.); amina.bari@usmba.ac.ma (A.B.); dalila.bousta@usmba.ac.ma (D.B.); 2 Department of Pharmaceutical Sciences, College of Pharmacy, Princess Nourah Bint Abdulrahman University, Riyadh 11564, Saudi Arabia; omalkmali@pnu.edu.sa; 3 Faculty of Arts and Sciences, Arab American University Palestine, Jenin 240, Palestine; hamada.imtara@aaup.edu; 4 Department of Chemistry, University of Helsinki, 00100 Helsinki, Finland; andriy.grafov@helsinki.fi

**Keywords:** antidepressant-like, anxiolytic, antioxidant, *Carum carvi* L., polyphenols, tail suspension test, light-dark chamber test, forced swim test, open field test, DPPH test, FRAP test

## Abstract

Depression and anxiety are widespread illnesses whose consequences on patients’ social and professional lives are becoming ever more dangerous and severe. The study’s objective is to explore the antidepressant-like and anxiolytic activity of the polyphenolic extract of *Carum carvi* L. as well as its antioxidant power as they were recently associated. The predictive antidepressant activity was evaluated using the forced swimming and tail suspension test in mice, a preclinical behavioral model widely used to determine the efficacy of antidepressant drugs. As for anxiolytic-like activity, two models were used, namely the light/dark chamber test to measure the animal’s degree of anxiety and the open field test to evaluate both anxiolytic and locomotor activity. The tests results indicate a remarkable antidepressant and anxiolytic-like effect after oral administration of the polyphenolic fraction of *C. carvi* and interesting antioxidant property. In the extract it has been confirmed the presence of 6 molecules belonging to polyphenols, identified with HPLC analysis. This study confirms and encourages the traditional use of the extract and appeals to further studies to understand its action mechanism.

## 1. Introduction

The 21st century represents a radical change for the human being in terms of lifestyles, rhythms, and demands. People are then subjected to new situations that they must face. As a result, many of them complain of anxiety, depression, or stress [1]. Alternative treatments currently have an important place in the therapeutic strategy [2].

The development of plant-derived anxiolytic and antidepressant drugs involves several investigations of ethnopharmacological, phytochemical, and pharmacological order. Moreover, the selection of a suitable plant for a pharmacological study is one of the most important steps and the various indices that should be taken into consideration include conventional application, chemical composition and toxicity [3].

About 50% of the therapeutic agents currently used come from natural sources, while less than 10% of plant species have been examined for their biological properties. These percentages indicate that new therapeutic discoveries can still be elucidated on medicinal plants [4].

Aromatic plants form a fundamental natural richness, for which valorization in its turn needs a perfect understanding of the physical and chemical properties which can be scientifically enhanced [5]. The properties of plants depend on the presence of various bioactive molecules belonging to different chemical families. 

Morocco, by its geographical situation, offers rich and diverse vegetation. It has about 4200 species, most of which are not exploited [6]. *Carum carvi* L., one of the Apiaceae family species, and considered to be one of the most used medicinal plants in conventional medicine, has been selected for this study [6]. The genus *Carum* is among the most popular genera in the Apiaceae family and has about 20–30 species from Europe, North Africa, and Asia [7]. This genus’s best-known species is *Carum carvi* L., Caraway seeds have been traditionally used as a condiment or spice thanks to their pleasant flavor [2].

It has also been used as an important source of spices and extracts with high antioxidant and antimicrobial power. This plant was also used to extract carvone, which is one of the main components of caraway essential oil, which inhibits the germination of stored potatoes and onions [8]. 

Many studies are currently interested in medicinal plants for their richness in secondary metabolites such as polyphenols, flavonoids, tannins, etc. They have a panacea of therapeutic virtues, including antioxidant and antimicrobial activities, anti-inflammatory, anticancer, and many others [9]. In this context, our work aims to evaluate the pharmacological activity of phenolic extracts of *Carum carvi* L. (PCC) in experimentally-induced anxiety and depression in mice as it’s been reported to be traditionally used for this purpose [10] and its correlation with the plant antioxidant properties using the DPPH (2,2-Diphenyl-1-picrylhydrazyl) and FRAP (Ferric reducing antioxidant power) test.

## 2. Results and Discussion

### 2.1. Extract Biochemical Analysis

**Figure 1:**
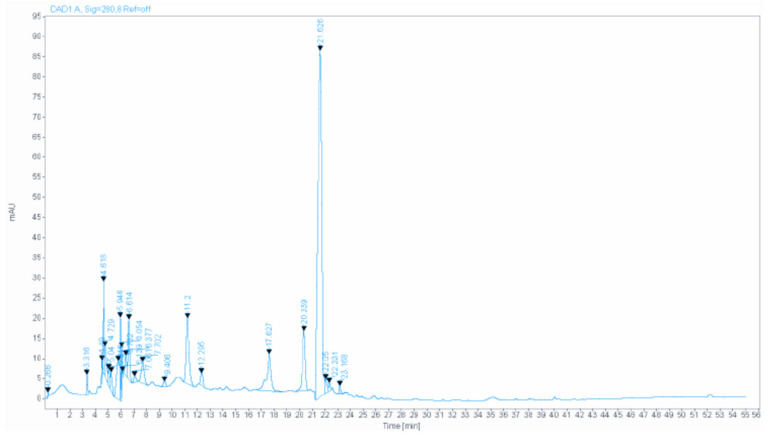
The HPLC chromatogram of PCC.

Figure 1 shows the HPLC (High-performance liquid chromatography) analysis chromatogram, and Table 1 represents the details of the main compounds. Almost all of the components identified were investigated for their anxiolytic and antidepressant-like effects, such as gallic acid [11], quercetin [12], catechin [13], myricetin [14], and caffeic acid [15].

Ferrulic acid antidepressant-like activity was assassed in different studies relating it to its antioxidant [16] and anti-inflammatory proprieties [17], with a strong promoting of metabolism energy, an increased neurotrophic, ATP and catecholamine levels and decreased level of glycogen in the limbic system [18]. This compound also demonstrated an anxiolytic effect in alcohol withdrawal induced anxiety in mice [19] suggesting it potential mecanism of action the partial involvement of the NMDA (N-methyl-D-aspartate) receptor [20].

### 2.2. Evaluation of the Anxiolytic Activity of the Polyphenolic Fraction of C. carvi

#### 2.2.1. Light and Dark Room

**Figure 2:**
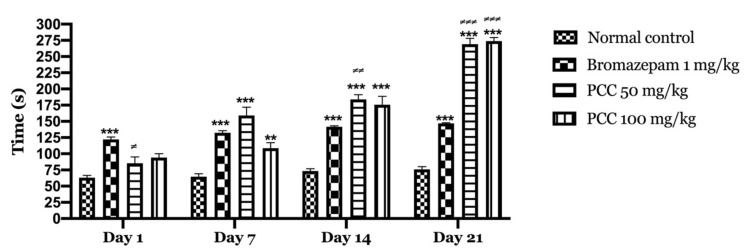
Time spent in the bright room in the light and dark room test. Time spent in the bright room in the light and dark room test. Data are displayed as mean ± SEM. A two-way analysis of variance (ANOVA) for repeated measures with Dunnett post hoc test were used to analyse the data. *: Represent the comparison of the different groups with the control (*** *p* < 0.001, ** *p* < 0.01) ≠: Represent the comparison of the different groups with bromazepam (≠≠≠ *p* < 0.001, ≠≠ *p* < 0.01, ≠ *p* < 0.05).

According to our results (Figure 2), bromazepam at 1 mg/kg significantly increased the time spent by the mice in the light compartment (122.2 ± 3.760 on day 1; 146.6 ± 1.068 on day 21) compared to the control group (63.2 ± 3.367 on day 1; 75.8 ± 4.352 on day 21). A significant increase in the time spent in the light compartment was observed with the administration of 50 and 100 mg/kg PCC of (85.4 ± 9.626 on day 1; 269 ± 9.055 on day 21) (94 ± 6.107 on day 1; 265 ± 7.21 on day 21) respectively compared to control.

**Figure 3:**
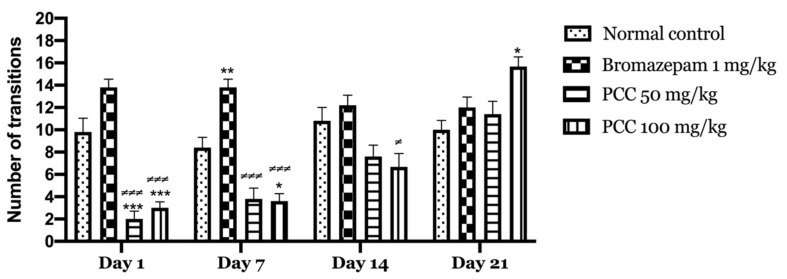
Number of transitions in the light and dark room test. Data are displayed as mean ± SEM. A two-way analysis of variance (ANOVA) for repeated measures with Dunnett post hoc test were used to analyse the data. *: Represent the comparison of the different groups with the control (*** *p* < 0.001, ** *p* < 0.01, * *p* < 0.05) ≠: Represent the comparison of the different groups with bromazepam (≠≠≠ *p* < 0.001, ≠ *p* < 0.05).

Figure 3 shows that the transitions of mice treated with PCC at the 50 mg/kg dose and those treated at the 100 mg/kg dose increase significantly (2.00 ± 0.707 on day 1; 11.40 ± 1.166 on day 21) (3.00 ± 0.548 on day 1; 16.00 ± 0.430 on day 21) respectively compared to the control (9.80 ± 1.241 on day 1; 10.00 ± 0.837 on day 21). A significant increase in transitions in treated mice with bromazepam 1 mg/kg (13.800 ± 0.735 on day 1; 12.00 ± 0.949 on day 21) was noted compared to control.

We noticed an increase in the time spent in the lighted chamber in mice treated with the PCC extract compared to the control. Besides, we recorded the increase in transition time compared to that of the control mice. This highlights the anxiolytic-like profile of the polyphenolic fraction of caraway.

To measure the anxiety level in mice in the light & dark room test, two parameters were used: the time spent in the dark room, which is proportional to the level of anxiety, and the number of transitions from one compartment to the other which is relatively proportional to the level of anxiolysis [21].

Based on the fact that an anxiolytic compound increases the time spent by the animal in the illuminated area, we assume that a mouse spending more time in the dark zone shows a higher anxiety level. An anxious animal will therefore spend more time in the dark area.

According to our results, a dose of 50 mg/kg of *C. carvi* polyphenolic extract exerts a significant anxiolytic-like effect. When the dose is increased to 100 mg/kg, we note that this effect is attenuated. This could be explained by sedation induced by this dose. Other studies have used the light/dark chamber test to evaluate the anxiolytic activity of the hydro-ethanolic extract of coriander at doses of 50, 100, and 200 mg/kg, the study results suggest that the extract produced an anxiolytic effect at 100 and 200 mg/kg [22].

A correlation between oxidative stress and mood disorders has been reported in recent studies [23,24] and psychological stress [25,26], This opens up new possibilities for anxiety and depression treatment and/or prevention, with the possible use of antioxidants. Desrumaux et al. [27] found that vitamin E deficiency in mice brains substantially improved the core markers of oxidative stress and anxiety-provoking activity in mice. Similarly, mice fed with vitamin C have recently demonstrated that this compound has an antidepressant effect in the tail suspension test [28]. The powerful antioxidant potential of polyphenols is well known [29] and their utilization could be an interesting approach to treat anxiety and depression. The anxiolytic activity of the polyphenolic fraction (PCC) can be attributed to our extract’s antioxidant activity. Numerous studies have shown that polyphenols are interesting natural antioxidant substances that can exert pharmacological effects on the central nervous system (CNS) [30,31]. Polyphenols exert partial agonist properties at benzodiazepine receptors, which gives them an impressive anxiolytic power [32].

#### 2.2.2. Open Field

**Figure 4:**
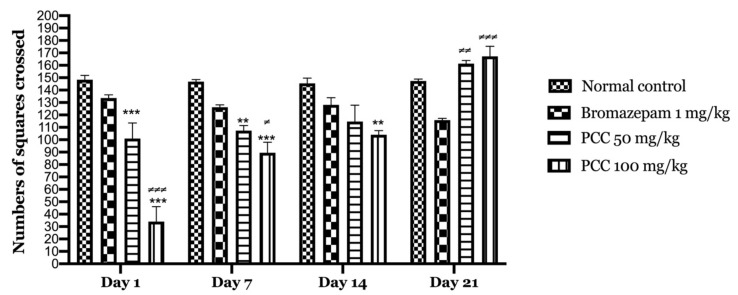
Numbers of squares crossed in the open field test. Data are displayed as mean ± SEM. A two-way analysis of variance (ANOVA) for repeated measures with Dunnett post hoc test were used to analyse the data. *: Represent the comparison of the different groups with the control (*** *p* < 0.001, ** *p* < 0.01) ≠: Represent the comparison of the different groups with bromazepam (≠≠≠ *p* < 0.001, ≠≠ *p* < 0.01, ≠ *p* < 0.05).

Our results show that downtime is significantly reduced in treated mice (PCC) compared to bromazepam and control group. Figure 4 shows that there is an increase in the number of tiles crossed in the PCC-treated groups at 50 mg/kg and 100 mg/kg of (100.8 ± 12.64 on day 1; 161.25 ± 15.705 on day 21) (34 ± 12.07 on day 1; 167.25 ± 10.39 on day 21) respectively compared to the control (148.4 ± 3.44 on day 1; 167.25 ± 10.39 on day 21).

**Figure 5:**
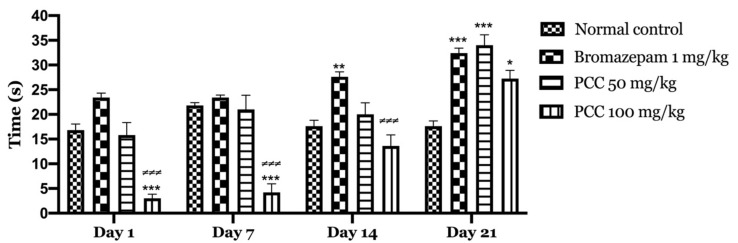
Time spent at the center in the open field test. Data are displayed as mean ± SEM. A two-way analysis of variance (ANOVA) for repeated measures with Dunnett post hoc test were used to analyse the data. *: Represent the comparison of the different groups with the control (*** *p* < 0.001, ** *p* < 0.01, * *p* < 0.05) ≠ Represent the comparison of the different groups with bromazepam (≠≠≠ *p* < 0.001).

We also find that the time spent in the center is significantly increased in treated mice (PCC) at 50 mg/kg and 100 mg/kg of (15.8 ± 2.551 on day 1; 34 ± 2.121 on day 21) (3 ± 0.837 on day 1; 27.25 ± 1.652 on day 21) respectively compared to the control (16.8 ± 1.241 on day 1; 17.6 ± 1.077 on day 21) (Figure 5).

We noted a significant increase in locomotion in the central part of mice treated with PCC extract at 50 mg/kg compared to the control. In addition, we recorded a decrease in locomotion in the peripheral part. Immobility time was decreased in mice treated with 50 mg/kg compared to the control mice.

In our study, we used the open-field test to assess anxiety in mice. Mice are nocturnal animals, accustomed to a dark and confined environment. However, this test’s center is open and bright, so it is the most anxious area for the animal in opposition to the periphery of the field that presents the least stressful area for the animal. As a result, control animals tend to spend more time in the periphery rather than in the center, which is considered the most anxiety-inducing zone [33].

An animal that is very active in the periphery and not very active in the center of the open field is considered to have a high anxiety level [34]. The variables measured to assess the animal’s anxiety level are: (i) the distribution of activity in the periphery and in the center, (ii) the time spent in each zone (periphery and center), indicate the anxiety level of the mice, and (iii) the number of recoveries indicating the level of its exploratory activity (habituation). Locomotion in the open field was evaluated by measuring the locomotion index in the peripheral part as well as in the central part as a function of time (total number of tiles).

Polyphenolic extract at 50 mg/kg and 100 mg/kg produced an anxiolytic effect due to the significant increase in time spent in the center and the number of tiles crossed. A study was performed by Mahendra et al. on the hydro-ethanolic extract of *Coriandrum sativum* at 50, 100, 200 mg/kg showed that the dose of 200 mg/kg has an anxiolytic-like activity. The latter is probably associated with its flavonoid content. The mechanism of action by which *C. sativum* exhibits anxiolytic-like activity may be similar to that of diazepam (which acts via the (GABA) receptor complex), because flavonoids and diazepam are structurally identical. The effects of flavonoids as anxiolytics have been observed in many plant species used in medicine [22].

### 2.3. Evaluation of the Antidepressant Activity of the Polyphenolic Fraction of C. carvi

#### 2.3.1. Forced Swimming

**Figure 6:**
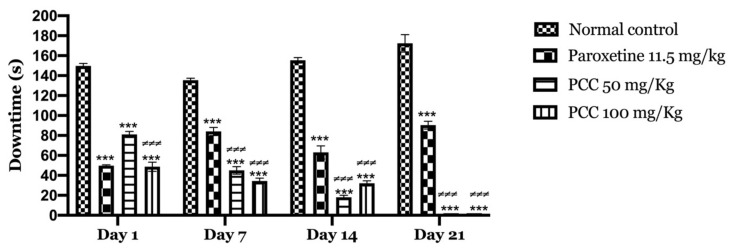
Downtime in forced swimming test. Data are displayed as mean ± SEM. A two-way analysis of variance (ANOVA) for repeated measures with Dunnett post hoc test were used to analyse the data. *: Represent the comparison of the different groups with the control (*** *p* < 0.001) ≠ Represent the comparison of the different groups with paroxetine (≠≠≠ *p* < 0.001).

Figure 6 shows the variation in immobility times, in control mice and mice treated with the polyphenolic extract of caraway at 50 and 100 mg/kg undergoing the forced swimming test. In our results, we found that the immobility time decreased significantly in mice treated with PCC at 50 and 100 mg/kg of (81 ± 3.055 on day 1; 0 ± 0.00 on day 21) (48.66 ± 4.631 on day 1; 0 ± 0.00 on day 21) compared to control mice (149.66 ± 2.603 on day 1; 172.33 ± 8.686 on day 21).

The forced swimming test is validated as an experimental approach to assess the potential efficacy of antidepressants in rodents and represents an aversive stressful situation from which the rat or mouse cannot escape, and produces immobility, i.e., behavioral despair [35]. Our results indicate an antidepressant effect of the polyphenolic extract of *C. carvi*, translated by a decrease in immobility time and with an increase in active behaviors, such as swimming and climbing. There is an excellent correlation between the predicted antidepressant activity in the FST assay and the clinically revealed antidepressant profile since 94% of the experimentally predicted antidepressant substances were future antidepressant molecules. This decrease in immobility may favor either an increase in swimming time or an increase in climbing time. Antidepressants that produce a predominantly noradrenergic and/or dopaminergic elevation reduce immobility by increasing the climbing time [36]. In contrast, those that reduce immobility by increasing swimming involve more central serotonin neurotransmission [37].

By comparing our results with those in the literature, researchers used the forced swimming test to evaluate the antidepressant activity of several plant extracts, for example, the methanolic extract of *Foeniculum vulgare* of the Apiaceae family. The results of this work showed that oral administration of *F. vulgare* extract produced antidepressant activity at a dose of 250 and 500 mg/kg in a dose-dependent manner compared to the control group [38]. Other studies on aqueous and ethanol extract of *Pimpinella anisum* of the Apiaceae family have shown that *P. anisum* has antidepressant activity at doses of 100 and 200 mg/kg in the forced swimming test [39].

The antioxidant activity of the polyphenolic extract of *Carum carvi* can be linked to PCC’s antidepressant activity. Studies have shown that polyphenols have similar effects to antidepressants [30,31] at lower doses than reference antidepressants. They can be used to stabilize the mood of depressed patients.

#### 2.3.2. The Tail Suspension Test

The polyphenolic extract of *Carum Carvi* L. showed an average decrease in immobility time on the first day of treatment, but decreased significantly after 7, 14 and 21 days of treatment.

The treated groups showed a considerable reduction in immobility time that was almost similar to that of standard paroxetine.

On day 1 of the test, the immobility time in the paroxetine, PCC 50 mg/kg and PCC 100 mg/kg groups were 99 ± 2.06, 123.8 ± 4.10 and 112 ± 2.81 s respectively, which was significant compared to the control group in which the immobility time was 161.67 ± 5.96 s.

On day 7 of the test, the immobility time in the paroxetine, PCC 50 mg/kg and PCC 100 mg/kg groups was 101.67 ± 2.62, 103.16 ± 2.45 and 100.33 ± 2.56 s respectively, which was significantly reduced compared to the control group in which the downtime time was 147.16 ± 4.95 s.

On day 14 of the test, the immobility time in the paroxetine, PCC 50 mg/kg and PCC 100 mg/kg groups was 92.33 ± 2.17, 102.33 ± 1.33 and 95.5 ± 4.04 s, respectively, which was also significantly reduced compared to the control (198.16 ± 5.95 s).

**Figure 7:**
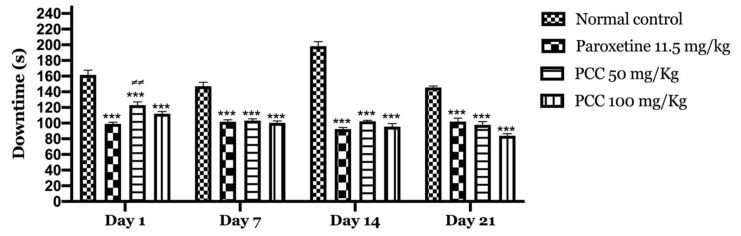
Downtime of the animals in the tail suspension test. Data are displayed as mean ± SEM. A two-way analysis of variance (ANOVA) for repeated measures with Dunnett post hoc test were used to analyse the data. *: Represent the comparison of the different groups with the control (*** *p* < 0.001) ≠: Represent the comparison of the different groups with paroxetine (≠≠ *p* < 0.01).

Similarly, on day 21 of the test, the immobility time in the paroxetine, PCC 50 mg/kg and PCC 100 mg/kg groups was 102 ± 4.45, 97.83 ± 4.19 and 83.83 ± 2.73 s, respectively, compared to 145.5 ± 1.99 s noted for the control group (Figure 7).

### 2.4. Antioxidant Activity

A compound’s antioxidant function refers to its capacity to prevent oxidation. Several aromatic plant extracts are considered an excellent source of antioxidant molecules. In this context, we evaluated the antioxidant activity of polyphenolic extract of caraway (seed), using DPPH and FRAP methods.

#### 2.4.1. DPPH (2,2-Diphenyl-1-picrylhydrazyl) Antioxidant Activity

The DPPH method is generally the most widely used method for rapid and direct assessment of antioxidant activity because of the free-radical form stability of DPPH and the simplicity of analysis. The polyphenolic extract’s antioxidant activity was evaluated using a methanolic solution of DPPH (0.004%). A dark purple color with optimum absorption at 517 nm is seen in the freshly prepared DPPH solution. When an antioxidant is active in the medium, this color normally disappears. Thus, DPPH free radicals can be neutralized by antioxidant molecules (by supplying hydrogen atoms or by contributing electrons) and transformed into a yellow-colored substance, resulting in a decrease in absorption.

**Figure 8:**
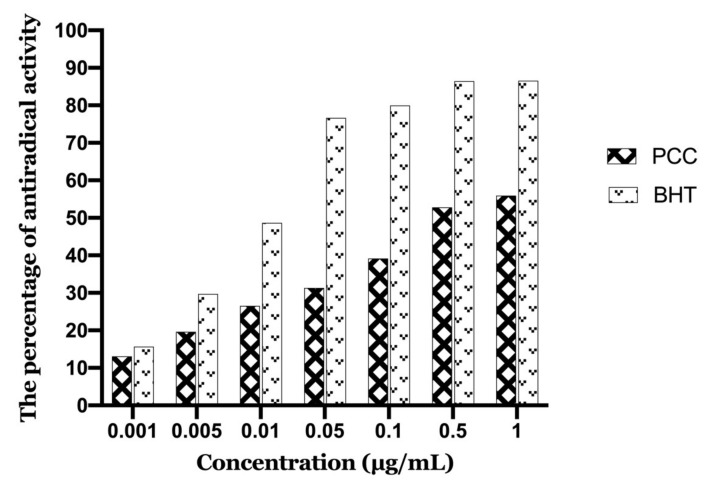
Antioxidant activity as a function of caraway polyphenol concentrations.

Figure 8 illustrates the evolution of antioxidant activity (%) of the polyphenolic extract studied, as well as the BHT (Butylated hydroxytoluene) used as a positive control. It seems that the percentage of free radical neutralization is proportional to the extract concentration.

The polyphenolic extract of caraway exerts antioxidant activity over the entire range of concentrations studied. Indeed, the concentration of 1 µg/mL inhibits 55.94% of DPPH. The concentration that provides 50% inhibition (IC50) was calculated from the calibration curve (PCC and BHT are 0.267 µg/mL and 0.024 µg/mL, respectively). Also, the lower the IC50 values, the stronger the antioxidant power. The IC50 value calculated for our PCC extract confirmed the reactivity of the sample to DPPH.

Compared to the standard antioxidant BHT (IC50 = 0.024 μg/mL) the PCC extract was less active. In general, for the plant studied, we have an interesting antioxidant activity (IC50 = 0.267 μg/mL).

#### 2.4.2. FRAP Method

It is a method of measuring our extract substances’ ability to reduce ferric iron (Fe^3+^) to ferrous iron (Fe^2+^). It is a fast, easy and reproducible technique [40].

The reducing capacity of a compound can serve as a significant indicator of its potential antioxidant activity. Previous work has indicated that there is a direct relationship between antioxidant activities and the reducing power of components in some plants [41]. An increase in absorbance corresponds to an increase in the tested extract’s reducing ability [42].

In our work, we tested by the FRAP method the polyphenolic extract of caraway with the positive control (BHT), whose absorbance was measured under the same conditions as the samples. The results obtained for iron reduction allowed us to draw curves (DO = f (concentration)) for each extract.

**Figure 9:**
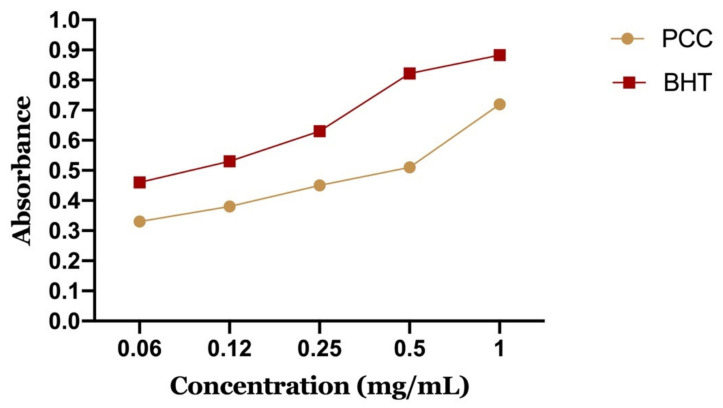
Reducing power of the polyphenolic fraction of *Carum Carvi* L.

Figure 9 shows the reducing power of PCC and BHT. It can be seen that the iron reduction capacity is proportional to the increase of the PCC concentration. The extract has a dose-dependent activity. However, PCC has a lower reducing power than BHT. We note that BHT has an important activity to reduce iron with a maximum optical density of 0.87 at a concentration of 1 mg/mL compared to PCC, which presented an OD of about 0.67 for the same concentration.

The 50% effective concentration (EC-50) was calculated from the calibration curve, which corresponds to the concentration of antioxidants required to obtain an absorbance of 0.5 nm. PCC represents a 50% effective concentration of 0.56 mg/mL and BHT of 0.09 mg/mL.

## 3. Materials and Methods

### 3.1. Extraction

*Carum carvi* L. seeds were collected from the region of Taounate (Fez, Morocco). The identification and authentication (BPRN15) was precisely done by a botanist, Professor Amina Bari at the laboratory of LBEAS, FSDM.

The vegetal material is delipidated in a 500 mL bottle, by adding 20 g of caraway powder to 200 mL of hexane (maceration for 1 h repeated three times). After filtration and drying, the residue obtained was re-extracted with 70% methanol in an ultrasound-assisted extraction apparatus (sonicator) for 40 min at a frequency of 35 kHz. After this, the extract was filtered using Whatman filter paper No. 5. The filtrate was dried and concentrated using a rotary evaporator.

The dry extract was diluted in distilled water and then washed out two times with chloroform and dichloromethane (v:v) to obtain a cleaner solution of the extract by getting rid of the left pigments and the mannitol. In this last step, the polyphenols found were concentrated and preserved for further use (final yield 2.7%).

### 3.2. Animals

Swiss albino mice were provided from the laboratory animal house (LBEAS) at the FSDM (Fez, Morocco). They were placed in groups of 5 in standard cages and subjected to 14 days adaptation period prior to the beginning of any experiment. During this time the animals had unrestricted access to food and water and were held at a steady temperature of 23 ± 2 °C and a 12/12 h light/dark cycle.

Two plant extract doses (50 and 100 mg/kg) were tested. Bromazepam (1 mg/kg) has been used as a positive control for anxiety tests, and paroxetine (11.5 mg/kg) for depression tests. The groups underwent 21 days of sub-acute treatment and were evaluated on the 1st, 7th, 14th, and 21st days [43]. The treatments were delivered orally to the animals by intragastric gavage using a gavage needle tipped with a stainless steel bulb to inject it into the stomach. The protocol (07#12/2019/LBEAS) was accepted by the institutional animal ethics committee.

### 3.3. Antidepressant Activity

#### Predictive Test for Depression in Animals: Porsolt Test or Forced Swimming Test

The forced swimming test or Porsolt’s model [44] is a pharmacological test predictive of a molecule’s antidepressant efficacy. This test is widely used to highlight the potential antidepressant properties of new molecules. It consists of forcing animals to swim in a closed enclosure at the height of 12 cm of water with no possibility of escape. This depth does not allow the animal to put its hind legs on the bottom of the cylinder. After a few minutes, the time spent swimming is reduced in favor of the immobility behavior, i.e., the animal floats passively, making only the movements necessary to keep its head out of the water. Immobility time is an indicator of the animal’s pseudo-depressed or resigned state. The administration of most conventional antidepressants reduces it. The total duration of the test is 6 min. A total of four batches of five mice each were used for this test, all products were administered by gavage 1 h before the start of the test.

### 3.4. Anxiolytic Activity

#### 3.4.1. Tail Suspension Test

The animal was hanged by its tail (2 cm from the end of the box (50 × 25 × 50 cm) for 6 min. When an animal has struggled to make any grappling action and hung with passivity without motion, it is called a suicidal activity and is noted as a time of immobility (only in the last 4 min were recorded for this test) [45].

#### 3.4.2. Open Field

It is used as a test of anxiety, exploration, and locomotion. We orally administered four groups (each with five animals), negative control (NaCl 9%), positive control treated with bromazepam (1 mg/kg), and two other groups treated with PCC at 50 and 100 mg/kg, once daily for 21 days was. After sixty minutes of treatment administration, the test was performed by placing each mouse in the central square to explore the arena, and a number of ambulations, rearings, and crossings of central squares were recorded using a digital video camera. The average number of total squares crossed by the mice and crosses of central squares is the number of times the mice entered the central squares with their four legs [46].

#### 3.4.3. Light and Dark Room

A wooden box of dimensions 44 cm × 21 cm × 21 cm is divided into two compartments, a black compartment with cover and a white compartment without cover. The two compartments were separated by a piece of wood with a hole of 7 cm × 7 cm in the center on the floor surface. The mice were divided into three groups (each consisting of five animals), received a dose of normal saline (9%), bromazepam (1 mg/kg) and PCC (50 and 100 mg/kg) once daily for 21 days. After 60 min of treatment, a 5-min test was performed by placing each mouse in the center of the black box, the time spent in each compartment was recorded by a digital video camera [47].

### 3.5. Antioxidant Activity

The extract tested antioxidant power was evaluated in vitro using two tests, the DPPH test and the reducing power test (FRAP).

#### 3.5.1. DPPH Test (1, 1-Diphenyl-2-picryhydrozyl)

The DPPH radical test is performed following the method described by Tepe in 2005 [48]. The test is performed by mixing 975 μL of a methanolic solution of DPPH (0.004%) with 100 μL of the sample. The optical density is estimated at 517 nm after being incubated at laboratory temperature for 30 min. For negative control, the sample is substituted by the methanol. The following Equation (1) determines the inhibition percentage of DPPH:

IP (%) = (A0 − A/A0) × 100(1)

IP: inhibition percentage; A0: the optical density of the free radical solution (DPPH) in the absence of the extract (negative control); A: absorbance of the free radical solution (DPPH) in the presence of the extract.

#### 3.5.2. Test of Iron-Reducing Power (FRAP)

This study was conducted using the Moattar technique [49]; 500 μL of phosphate buffer (0.2 M; pH = 6.6) and 500 μL of 1% potassium ferricyanide (K_3_Fe(CN)_6_) are added to 100 μL of the various concentrations of the prepared samples in methanol. After 20 min of incubation in a water bath at 50 °C, 500 μL of 10% TCA solution, 100 μL of FeCl_3_ (0.1%) and 0.5 mL of distilled water are added to the reaction medium. In the absence of the test sample, absorbance is then measured at 700 nm against a blank comprising all the reagents. The findings are expressed as a 50 percent effective concentration (EC-50) representing the antioxidant concentration needed to reach an absorbance of 0.5 nm.

### 3.6. Extract HPLC Analysis

For the *C. carvi* extract analysis, An 1260 infinity II high-performance liquid chromatography (HPLC) system (Agilent Technologies, Santa Clara, CA, USA) was used. Fitted with a quaternary pump, the apparatus was coupled with a UV detector. The mobile phase was composed of two solvents, A: 0.1 percent acidified water and B: acetonitrile. A C18 Zorbax eclipse plus C_18_ column (5 μm, 4.6 × 150 mm) was used for the separation. The temperature of the column furnace was set at 35 °C. The sample injection volume and flow rates were set at 10 μL (100 μg/mL of sample concentration) and 1 mL/min. The concentration was calculated according to the following Equation (2), depending on the retention time (RT) and spectral correspondence of each compound:

Concentration (μg/mL) = (Area (sample)/area (standard)) × 100(2)

### 3.7. Statistical Analysis of Results

The results of the analysis were expressed using the program Graph Pad Prism 7 for Windows (Microsoft, Albuquerque, NM, USA) as mean ± standard error of the mean (SEM). A two-way variance analysis (ANOVA) followed by a Dunnett post hoc test was used to conduct data analysis. Significant results were considered when *p* < 0.05.

## 4. Conclusions

Conventional treatments used to treat many forms of anxiety and depression have undesirable side effects. Several alternative treatment strategies remain of crucial importance. In this context, medicinal plants are a good source for discovering new remedies for these disorders. In the search for an alternative therapy that is inexpensive and free of side effects, this research has been conducted to study the natural anxiolytic and antidepressant effect of the caraway polyphenolic fraction in anxious and pseudo depressed mice. Our results suggest that the PCC, administered orally, exerts interesting anxiolytic and antidepressant-like effects, particularly at the dose of 50 mg/kg. This study also encourages further studies to reveal the exact mode of action and the limitation of use of this plant.

## Figures and Tables

**Table 1:** The HPLC result of PCC.

Extract	Molecules	Retention Time	Retention Time (Standard)	Concentration (µg/mL)
PCC	Ferulic acid	21.020	21.039	27.70
	Gallic acid	6.614	6.645	9.17
	Myricetin	22.231	22.278	7.50
	Catechin	12.295	12.777	6.80
	Caffeic acid	17.627	17.336	6.77
	Quercetin	23.168	23.350	4.77

## Data Availability

Data are available upon reasonable request.
